# The Effect of Alignment Changes on Unilateral Transtibial Amputee’s Gait: A Systematic Review

**DOI:** 10.1371/journal.pone.0167466

**Published:** 2016-12-06

**Authors:** Niels Jonkergouw, Maarten R. Prins, Arjan W. P. Buis, Peter van der Wurff

**Affiliations:** 1 Department of Orthopaedic Technology, Military Rehabilitation Centre Aardenburg, Doorn, the Netherlands; 2 Department of Biomedical Engineering, University of Strathclyde, Glasgow, Scotland, United Kingdom; 3 Department of Research and Development, Military Rehabilitation Centre Aardenburg, Doorn, the Netherlands; 4 Research Institute MOVE, Faculty of Human Movement Sciences, VU University, Amsterdam, the Netherlands; 5 Department of Physical Therapy, HU University of Applied Sciences Utrecht, the Netherlands; Northwestern University, UNITED STATES

## Abstract

**Introduction:**

Prosthetic alignment, positioning of a prosthetic foot relative to a socket, is an iterative process in which an amputee’s gait is optimized through repetitive optical gait observation and induction of alignment adjustments when deviations are detected in spatiotemporal and kinematic gait parameters. An important limitation of the current prosthetic alignment approach is the subjectivity and the lack of standardized quantifiable baseline values. The purpose of this systematic review is to investigate if an optimal alignment criterion can be derived from published articles. Moreover, we investigated the effect of alignment changes on spatiotemporal, kinematic and kinetic gait parameters.

**Results:**

A total of 11 studies were included, two controlled before-and-after studies and nine-interrupted time series studies.

**Discussion:**

The results demonstrate that alignment changes have a predictable influence on the included kinetic parameters. However, the effect of alignment changes on spatio-temporal and kinematic gait parameters are generally unpredictable. These findings suggest that it is imperative to include kinetics in the process of dynamic prosthetic alignment. Partially this can be established by communication with the prosthetic user in terms of perceived socket comfort, but the use of measurement tools should also be considered. While current literature is not conclusive about an optimal alignment, future alignment research should focus on alignment optimisation based on kinetic outcomes.

## Introduction

Individuals with a transtibial amputation (TTA) have an altered functioning ankle joint, resulting in the loss of important ambulation functions, including energy absorption, propulsion and active push-off. To compensate for the functional loss, the human body adapts so that it can perform the necessary functions despite the structural asymmetry between the prosthetic limb (PL) and the non-prosthetic limb (NPL) [1].

During prosthetic fitting, the prosthetist will examine both the socket fit and the prosthetic alignment. Prosthetic alignment involves altering the position of a prosthetic foot relative to a socket. This process aims to reduce an amputee’s restrictions and discomfort during daily life activity [2]. By changing the relative orientation of the foot in respect to the socket, a prosthetist is able to influence spatiotemporal, kinematic and kinetic gait parameters in order to optimize the dynamic balance and biomechanical function exhibited by the user [3]. One aspect of the alignment process is dynamic alignment, where possible gait deviations are observed in a clinical environment. Adjustments of the position and orientation of the foot relative to the socket are applied in the sagittal-, coronal- and transverse-plane, so that the amputee’s gait is influenced to achieve minimal optical gait deviations and to satisfy both user and prosthetist with the resulting gait.

One limitation of dynamic alignment is its attempt to achieve a perfectly ‘normal’ symmetrical gait. Firstly, the prosthetic user’s personal limitations may require gait deviations. Secondly, increasing the symmetry in TTA gait does not necessarily result in gait improvement [4]. Multiple studies reported dissimilarities between TTA gait and nondisabled gait. Lower average cadence, velocity, stride length and single limb support time are observed in TTA gait [5–8]. Additionally, the swing time and step length of the PL are found to be greater, and the stance phase duration on the NPL are increased, compared to the PL [9,10]. Asymmetry may be a prerequisite to achieve gait stability during ambulation [1]. Therefore, it may be argued that kinematic and spatiotemporal symmetry should not be the goal of dynamic alignment of the prosthesis.

A more extensive and objective interpretation of the effect of different prosthetic alignments on the gait pattern of TTA patients is desirable in an attempt to define optimal alignment. The current interpretation of optimal alignment is subjective, with both the prosthetist and user indicating optimal gait through a trial-and-error process. As a result, the dynamic alignment achieved by the conventional dynamic alignment process is found to be highly variable, with a wide range of alignments tolerated by below knee prosthetic users [11]. Although the prosthetist may observe an optimal gait after alignment alterations, the prosthetic user may actually be maintaining the given gait pattern with strongly altered kinetics. Detailed examination of the generated joint moments required to produce the observed gait may reveal the abnormalities [8]. The relationship between prosthetic adjustments, gait deviations and compensatory mechanisms remain unclear.

This systematic review will examine the current literature of alignment changes on spatiotemporal, kinematic and kinetic parameters in TTA gait. Secondly, the review will attempt to find the most widely accepted definition of optimal alignment for TTA gait.

## Methods

**Fig 1:**
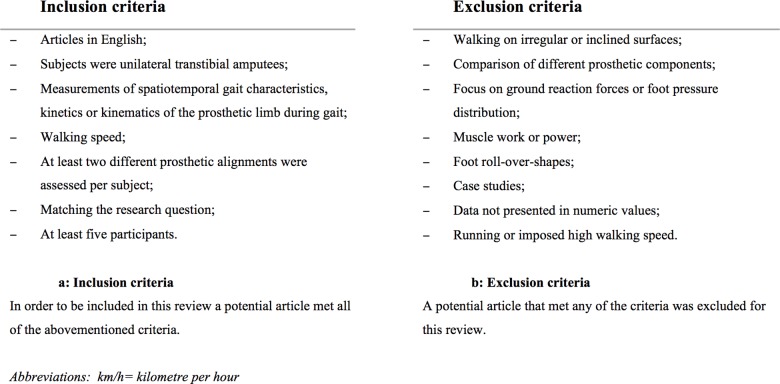
Inclusion and exclusion criteria.

Our study was conducted and reported according to the Preferred Reporting Items for Systematic Reviews and Meta-analysis Statement (S1 Checklist). A systematic search was performed in the PubMed and Embase databases, from the initiation of the databases up to October 15^th^, 2014 (Table 1). Two reviewers, (NJ and PvdW) independently screened each title to select potentially relevant papers. The resulting abstracts were screened to select articles matching the research question (Fig 1). Only articles fulfilling both the in- and exclusion criteria following a full text review were included in the systematic review. The references of the final selection of articles were reviewed to find potentially relevant articles, which may have been missed, in the initial search.

**Table 1:** Search strategy.

Database	Search Strategy	n-hits
PubMed search syntax	((“Trans-tibial” [Title/Abstract] OR Transtibial [Title/Abstract] OR “Below-knee” [Title/Abstract]) AND (Amput* [Title/Abstract])) AND ((“Alignment” [Title/Abstract] OR “Malalignment” [Title/Abstract] OR “Align” [Title/Abstract]) AND (Prosthe* [Title/Abstract])).	**72**
EMBASE search syntax	((‘Trans-tibial’:ti:ab OR Transtibial:ti:ab OR ‘Below-knee’:ti:ab) AND (Amput*:ti:ab)) AND ((‘Alignment’:ti:ab OR ‘Malalignment’:ti:ab OR ‘Align’:ti:ab) AND (Prosthe*:ti:ab))	**75**
Total		**147**

Data extracted from the studies included: study design, population (sample size and age), initial prosthetic alignment, prosthetic alignment adjustments, all reported spatiotemporal gait parameters and kinematics and kinetics of both lower extremities during gait.

To the best of the authors’ knowledge, no validated critical appraisal has been published which matches this review’s scope. As such, an adapted version of a non-validated critical appraisal by van der Linde *et al*. was used [12]. Van der Linde *et al*. based their appraisal on the integration of two existing criteria lists for quality assessments of randomized controlled trials [13,14]. Each of those criteria were individually assessed and adapted to the relevance of this systematic review, for both before-and-after study designs and interrupted time series designs. Some criteria were removed or altered where they scored ‘0’ or ‘1’ by definition. The adapted critical appraisal evaluated a checklist of ten criteria (S1 Appraisal).

To determine the risk of bias in this review we deviated from the Cochrane system because this is primarily designed for intervention studies. Instead, we utilized the COSMIN system box F: ‘Hypothesis testing’, to evaluate the risk of bias per studied measurement property [15,16]. Each item is scored on a four-point scale (Excellent, Good, Fair or Poor) and the final score is judged by using a ‘worst count score’. In accordance with previous studies we relinquish from judgements on sample size requirements in this quality assessment procedure because it was expected that all studies contains less than 30 subjects [15]. Two reviewers independently scored the included articles; any dissimilarity in an item assessment was resolved by a consensus meeting.

## Results

**Fig 2:**
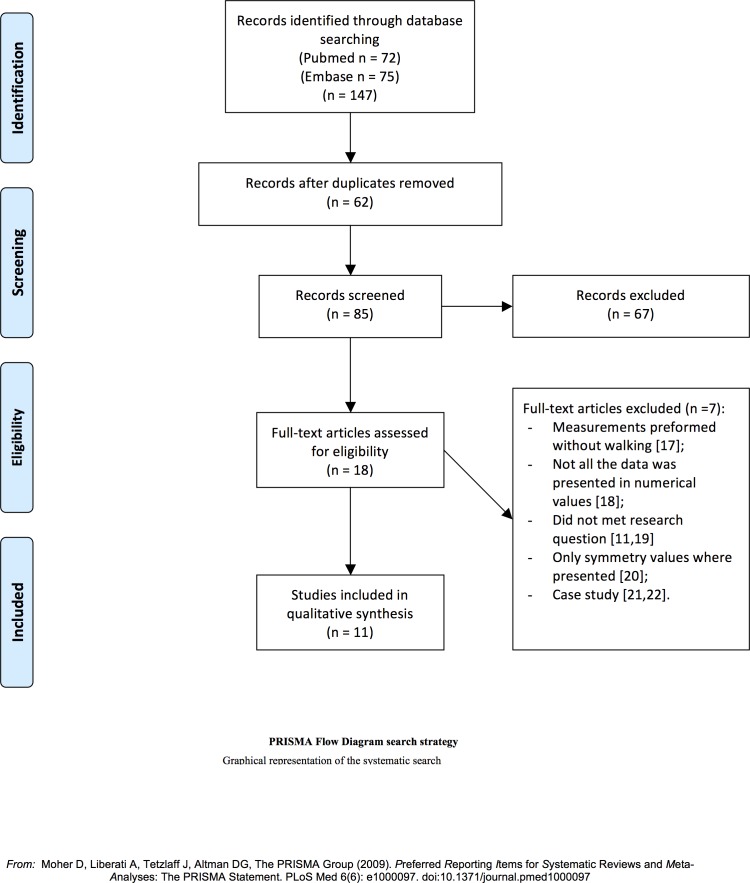
PRISMA flow diagram.

A total of 85 studies were identified using the search strategy as shown in Fig 2. After full text assessment 7 studies were excluded [11,17–22] Followed by screening of the reference section of the remaining 11 studies, this did not result in the inclusion of additional studies. The studies included in this review include two controlled before-and-after study design studies and nine-interrupted time series design studies (Table 2).

**Table 2:** Studies included in systematic review.

First author	Cohort	Research focus	N	Sex (F/M)		Age mean (SD) year	Prosthetic Feet	Time since amputation Mean (SD) year	Study Design	Selection			Intervention						Statistical Validity				Total Score	Hypothesis testing exclusive of sample size*
										A1	A2	A	B3	B4	B 5A	B 5B	B6	B	C7	C8	C9	C		Box F
Beyaert [24]	Centre de Readaptation Louis Pierquin Cohort, 2008	Compensatory function of the knee joint of the NPL related to transverse prosthetic foot alignment perturbation.	15 AB		NR	46 (16)	2 SACH	16.7 (17.6)	CBA	0	0	0	1	1	1	1	1	5	1	1	1	3	**8**	**Fair**
Grumillier [23]		Compensatory hip function in response to uncomfortable gait induced by transverse prosthetic foot alignment.	17 TTA		NR	45 (17)	15 ESR		CBA	0	0	0	1	1	1	1	1	5	1	1	1	3	**8**	**Fair**
Boone [30]	Orthocare Innovation cohort, 2012	Effect of trans-tibial alignment changes on the moments measured at the base of the socket.	11 TTA		1F/ 10M	47 (13)	11 SACH	NR	ITS	0	0	0	1	1	0	0	1	3	1	1	1	3	**6**	**Good**
Kobayashi [31]		The effect of systematic coronal alignment changes on sagittal socket reaction moments and vice versa, with trans-tibial amputees.							ITS	0	0	0	1	1	0	1	1	4	1	1	1	3	**7**	**Good**
Kobayashi [27]		The effect of alignment perturbations on sagittal and coronal socket reaction moment interactions.							ITS	0	0	0	1	1	0	1	1	4	1	1	0	2	**6**	**Fair**
Kobayashi [28]		Individual response to alignment perturbations by measurement of the external socket reaction moment.							ITS	0	0	0	0	1	0	0	1	2	1	1	1	3	**5**	**Fair**
Fridman [25]	Unique cohort	Influence of transverse prosthetic foot alignment on gait parameters and compensating patterns.	8 TTA		8M	49 (9)	8 SACH	13.5 (10.0)	ITS	0	1	1	0	0	1	1	1	3	0	1	1	2	**6**	**Fair**
Kobayashi [26]	Orthocare Innovation cohort, 2014	Effect of alignment changes on the external socket reaction moment while using energy storage and return feet.	10 TTA		4F/ 6M	50 (11)	10 ESR	17 (14)	ITS	0	0	0	1	1	0	1	1	4	1	1	1	3	**7**	**Good**
Kobayashi [33]		Effect of alignment changes on the socket reaction moment impulse, in the plane of the adjustment.							ITS	0	0	0	1	1	0	1	1	4	1	1	1	3	**7**	**Good**
Pinzur [29]	Unique cohort	Investigate the relationship between prosthetic alignment and tentative load on the PL and on the NPL of unilateral amputees	14 TTA		2F/ 12M	45 (NR)	14 SACH	NR	ITS	0	0	0	0	1	0	0	1	2	1	0	0	1	**3**	**Fair**
Schmalz [32]	Unique cohort	Defining the influence of prosthetic alignment on the metabolic energy consumption and biomechanical gait parameters during walking.	7 TTA		NR	49 (17)	7 SACH	23 (19)	ITS	0	0	0	0	0	1	0	0	1	0	1	1	2	**3**	**Fair**

Studies that reported data of one and the same cohort are subdivided into those cohorts. Every article was scored on three criteria: Intervention, Statistical Analysis and Validity and scored on methodological quality by the COSMIN checklist, Box F: Hypothesis testing.

Abbreviations: AB = Abled Bodied, CBA = Controlled Before-and-After Study, ESR = Energy Storage and Return feet, F = Female, ITS = Interrupted Time Series Designs, M = Male, NPL = Non Prosthetic Leg, NR = Not Reported, PL = Prosthetic Leg, SACH = Solid Ankle Cushion Heel, SD = Standard Deviation, TTA = Transtibial Amputee

* Methodological quality by COSMIN guideline for systematic reviews.

Four studies scored a good and seven studies a fair for the hypotheses assessment by the COSMIN guideline. The critical appraisal showed criterion specific shortcomings, which are mainly present in the selection criteria. Due to a lack of descriptive data the homogeneity of the investigated groups is questionable. The quality assessments of all the included studies are listed in Table 2.

Of the included studies, three compared gait parameters of the NPL with the PL in unilateral TTA [23–25], whereas the other eight only investigated the PL [26–33] Of the three studies examining both the NPL and the PL, two included an additional group of abled-bodied persons for comparison [23,24]. The relevant outcomes are subdivided into three result tables: spatiotemporal (Table 3), kinematic (Table 4) and kinetic (Table 5) data.

**Table 3:** Spatiotemporal gait parameters.

First author(s)	Cohort	Outcome variable	Value nominal alignment (SD)	Condition							
				Value (SD)							
	Centre de Readaptation Louis Pierquin Cohort, 2008			+6° IR		+6° ER		** **		** **	
Beyaert [24]		Walking speed (m/s)	1.36 (0.20)	1.35 (0.18)		1.34 (0.20)		** **			
Grumillier [23]		Cadence (steps/min)	109 (8)	109 (8)		108 (8)					
		Stride length (m)	1.51 (0.19)	1.50 (0.18)		1.47 (0.19)					
		Single support phase PL (s)	0.42 (0.03)	0.40 (0.04)*^+^		0.41 (0.04) ^+^					
		Single support phase NPL (s)	0.44 (0.03)	0.44 (0.03) ^+^		0.44 (0.03) ^+^					
		Stance phase PL (% of GC)	61 (2)	60 (1) ^+^		61 (2) ^+^					
		Stance phase NPL (% of GC)	63 (2)	64 (2)*^+^		64 (2)*^+^					
Kobayashi [27]	Orthocare Innovation cohort, 2012	** **		Flexion		Extension		Abduction		Adduction	
Kobayashi [28]				3°	6°	3°	6°	3°	6°	3°	6°
Kobayashi [31]		Cadence (steps/min)	109 (16)	NR	104 (13)*	101 (17)*	100 (15)*	NR	NR	103 (19)*	NR
		Stance phase (% of GC) max. varus/valgus moment occurred	31	33	29	27	26	31	32	14	18
		Stance phase (% of GC) max. extension moment occurred	76	76	77	73	73	76	76	75	75
				Anterior translation		Posterior translation		Medial translation		Lateral translation	
				5mm	10mm	5mm	10mm	5mm	10mm	5mm	10mm
		Cadence (steps/min)	109 (16)	NR	NR	NR	NR	NR	NR	NR	NR
		Stance phase (% of GC) max. varus/valgus moment occurred	31	28	30	30	25	29	15	32	31
		Stance phase (% of GC) max. extension moment occurred	76	76	75	74	74	76	74	76	75
	Unique cohort		** **	+18° ER		+36° ER					
Fridman [25]		**Stance time** on PL (s)	0.77 (0.08)	0.75 (0.09)		0.73 (0.09)					
		Stance time on NPL (s)	0.78 (0.09)	0.78 (0.11)		0.79 (0.11) ^+^					
		Inter leg stance time difference (s)	0.02 (0.05)	0.03 (0.05)		0.06 (0.07)*					
		Leg symmetry ratio (NPL vs. PL)	0.98 (0.06)	0.97 (0.06)		0.92 (0.08)*					
	Unique cohort	**Swing time** of PL (s)	0.43 (0.05)	0.44 (0.04)		0.44 (0.05)					
		Swing time of NPL (s)	0.41 (0.04)	0.41 (0.03)		0.39 (0.04) ^+^					
*Continues from previous page*		Inter leg swing time difference (s)	0.02 (0.04)	0.03 (0.03)		0.05 (0.04)*					
		Leg symmetry ratio (NPL vs. PL)	0.952 (0.09)	0.937 (0.08)		0.893 (0.08)*					
		**Step length** of PL (cm)	67.36 (10.26)	66.76 (10.07) ^+^		65.10 (10.97) ^+^					
		Step length of NPL (cm)	63.06 (7.08)	62.13 (7.79) ^+^		59.63 (8.45) ^+^					
		Inter leg step length difference (cm)	3.16 (4.93)	4.61 (5.58)		5.83 (5.12)*					
		Leg symmetry ratio (NPL vs. PL)	0.96 (0.07)	0.94 (0.08)		0.92 (0.07)*					
Kobayashi [26]	Orthocare Innovation cohort. 2014	Stance time (s)	0.72 (0.11)	No significant change in all 24 malaligned conditions compared to nominal alignment							
Kobayashi [33]		Cadence (steps/min)	104 (12)	No significant change in all 24 malaligned conditions compared to nominal alignment							
Pinzur [29]	Unique cohort			+10° Adduction		+10° Abduction		+10° Flexion		+10° Extension	
		Stance time (s)	0.83 (9.84 (SEM))	0.83 (9.84 (SEM))		0.87 (9.84 (SEM))		0.87 (9.84 (SEM))		0.84 (9.84 (SEM))	
Schmalz [32]	Unique cohort			20mm Posterior translation		20mm Anterior translation		+10 dorsiflexion		+10 plantar flexion	
		Walking speed (km/h)	5.2 (0.6)	5.2 (0.4)		5.1 (0.5)		5.1 (0.4)		5.0 (0.6)	
		Stride length (m)	0.73 (0.05)	0.75 (0.05)		0.73 (0.04)		0.73 (0.05)		0.74 (0.06)	

Abbreviations: cm = centimetre, ER = External Rotation, GC = Gait Cycle, IA = Initial Alignment, IR = Internal Rotation, km/h = kilometre a hour, m = metre, mm = millimetre, m/s = metre per second, min. = minute, NPL = Non Prosthetic Limb, NR = Not Reported, N/S = Not Significant, PL = Prosthetic Limb, s = seconds, SEM = Standard error of the mean

* Significant difference (p<0,05) between nominal alignment and malaligned condition

+ Significant difference (P<0,05) between PL and NPL in the same condition

**Table 4:** Kinematic gait parameters.

First author(s)	Cohort	Outcome variable	Value nominal alignment (SD)	Condition			
				Value (SD)			
Beyaert [24]	Centre de Readaptation Louis Pierquin Cohort, 2008	**Foot (**- = external rotation)	* *		Control group	+6° IR	+6° ER
		Foot angle PL (°)	*-3 (5*)		-8 (4)	*7 (8*) *	*-12 (6*) *
Grumillier [23]		Foot angle NPL (°)	*-8 (9)*		-8 (4)	*-8 (9)*	*-9 (9)*
		**Knee (***- = extension)*	* *				
		Max. Knee flexion PL (°)	*15 (9*)		19 (4)	*10 (8*) *^+^	*14 (8*) ^*+*^
		Max. Knee flexion NPL (°)	*24 (7*)		19 (4)	*28 (7*) *^+^	*23 (9*) ^*+*^
		**Hip RoM (***- = extension)*	* *				
		Sagittal RoM of PL during 0–8% of GC (°)	*-4*.*9 (1*.*7*) ^*+*^ ^*$*^		-2.3 (1.6)	*-5*.*0 (1*.*8*) ^*+*^^*$*^	*-4*.*2 (1*.*5*) ^*+*^ ^*$*^
		Sagittal RoM of NPL during 0–8% of GC (°)	*3*.*0 (1*.*9*) ^*+*^^*$*^		-2.3 (1.6)	*4*.*6 (2*.*5*) **^+^ ^*$*^	*3*.*2 (2*.*5*) ^*+*^ ^*$*^
		Sagittal RoM of PL during 8–30% of GC (°)	*-22*.*2 (4*.*8)*		-21.3 (4.0)	*23*.*1 (5*.*2)*	*-21*.*7 (4*.*2)*
		Sagittal RoM of NPL during 8–30% of GC (°)	*-27*.*8 (3*.*0*) ^*+*^ ^*$*^		-21.3 (4.0)	*-28*.*3 (4*.*8*) ^*+*^ ^*$*^	*-26*.*7 (4*.*1*) ^*+*^ ^*$*^
		**Hip Flexion** (- = extension)	* *				
		Max. Hip Flexion occurred at PL (*%* of GC)	*92*.*7 (4*.*0)* ^*+*^ ^*$*^		91.7 (4.0)	*92*.*5 (4*.*1)* ^*+*^ ^*$*^	*92*.*6 (4*.*6)* ^*+*^ ^*$*^
		Max. Hip Flexion occurred at NPL (*%* of GC)	*7*.*8 (1*.*3)* ^*+*^ ^*$*^		91.7 (4.0)	8.1 (1.3) ^*+*^ ^*$*^	*8*.*5 (1*.*9)* ^*+*^ ^*$*^
		Max. Hip Flexion at NPL (°)	41.1 (6.7)		38.8 (4.6)	42.7 (5.8) **^$^	*41*.*1 (6*.*9)*
						
Fridman [25]	Unique cohort	**Foot** (- = external rotation)			^*+*^18° ER	^*+*^36° ER	
		Foot rotation PL (°)	*-10*.*9 (5*.*2)*		*-23*.*2 (6*.*3*) *^+^	*-37*.*1 (9*.*9*) *^+^	* *
		Foot rotation NPL (°)	*-13*.*9 (8*.*1)*		*-14*.*4 (7*.*0*) ^*+*^	*-12*.*1 (9*.*4*) ^*+*^	* *
		Inter-leg difference (°)	*3*.*2 (6*.*8*)		*9*.*1 (7*.*4*) *	*23*.*8 (13*.*5*) *	*** ***

Abbreviations: cm = centimetre, ER = External Rotation, GC = Gait Cycle, IR = Internal Rotation, NPL = Non Prosthetic Limb, PL = Prosthetic Limb, RoM = Range of Motion, SD = Standard Deviation

* Significant difference (p<0.05) between nominal alignment and malaligned condition

** Significant difference (p<0.01) between nominal alignment and malaligned condition

^**+**^ Significant difference (P<0.05) between PL and NPL in the same condition

^$^ Significant difference (P<0.05) between PL and control group. Only applicable for Beyaert et al. [16] and Grumillier et al. [15].

**Table 5:** Kinetic gait parameters.

First author	Cohort	Outcome variable	Value nominal alignment	Condition											
			Mean (SD)	Mean (SD)											
	Orthocare Innovation cohort, 2012			Flexion			Extension			Anterior translation			Posterior translation		
Kobayashi [27]		**Sagittal (- = flexion)**		3°		6°	3°		6°	5mm		10mm	5mm		10mm
Kobayashi [28]		Minimum moment: (Nm/kg)	-0.147 (0.117)	-0.104 (0.116)**		-0.077 (0.120)**	-0.144 (0.116)		-0.158 (0.106)	-0.163 (0.131)		-0.187 (0.134)	-0.095 (0.112)**		-0.059 (0.120)**
Boone [30]		Moment at 45% of stance phase: (Nm/kg)	0.217 (0.141)	0.176 (0.177)		0.160 (0.182)	0.363 (0.125)**		0.369 (0.129)*	0.227 (0.129)		0.203 (0.136)	0.252 (0.118)		0.287 (0.152)
Kobayashi [31]		Maximum moment: (Nm/kg)	0.719 (0.177)	0.755 (0.157)		0.793 (0.142)	0.672 (0.183)		0.609 (0.223)**	0.693 (0.153)		0.613 (0.153)**	0.776 (0.153)*		0.821 (0.149)**
				Abduction			Adduction			Medial translation			Lateral translation		
				3°		6°	3°		6°	5mm		10mm	5mm		10mm
		Minimum moment: (Nm/kg)	-0.152 (0.112)	-0.128 (0.109)		-0.142 (0.111)	-0.137 (0.114)		-0.142 (0.118)	-0.119 (0.098)		-0.132 (0.117)	-0.122 (0.131)		-0.137 (0.130)
		Moment at 45% of stance phase: (Nm/kg)	0.217 (0.141)	0.248 (0.133)		0.269 (0.127)	0.255 (0.172)		0.249 (0.180)	0.244 (0.121)		0.304 (0.212)	0.250 (0.174)		0.217 (0.105)
		Maximum moment: (Nm/kg)	0.719 (0.177)	0.726 (0.165)		0.722 (0.162)	0.708 (0.176)		0.729 (0.174)	0.730 (0.152)		0.718 (0.139	0.722 (0.175)		0.735 (0.194)
				Flexion			Extension			Anterior translation			Posterior translation		
		**Coronal plane (- = varus)**		3°		6°	3°		6°	5mm		10mm	5mm		10mm
		Moment at 30% of the stance phase: (Nm/kg)	-0.077 (0.078)	-0.073 (0.079)		-0.072 (0.073)	-0.033 (0.075)*		-0.029 (0.071)*	-0.067 (0.086)		-0.055 (0.076)	-0.060 (0.067)		-0.072 (0.073)
		Moment at 75% of the stance phase: (Nm/kg)	0.013 (0.055)	0.001 (0.065)		0.003 (0.063)	-0.006 (0.068)		-0.019 (0.063)	0.010 (0.076)		0.002 (0.061)	0.016 (0.075)		0.021 (0.088)
				Abduction			Adduction			Medial translation			Lateral translation		
				3°		6°	3°		6°	5mm		10mm	5mm		10mm
		Moment at 30% of the stance phase: (Nm/kg)	-0.077 (0.078)	-0.013 (0.077)**		-0.017 (0.066)**	-0.006 (0.073)**		0.074 (0.078)**	-0.033 (0.086)**		0.025 (0.069)**	-0.109 (0.082)**		-0.016 (0.086)**
		Moment at 75% of the stance phase: (Nm/kg)	0.013 (0.055)	-0.046 (0.090)*		-0.089 (0.110)**	0.049 (0.073)*		0.111 (0.085)**	0.054 (0.068)*		0.096 (0.060)**	-0.023 (0.073)**		-0.062 (0.084)**
				Flexion			Extension			Anterior translation			Posterior translation		
Kobayashi [26]	Orthocare Innovation cohort, 2014	**Sagittal (- = flexion)**		2°	4°	6°	2°	4°	6°	5mm	10mm	15mm	5mm	10mm	15mm
		Extension moment impulse: (Nm·s/kg)	0.166 (0.051)	0.170 (0.055)	0.173 (0.062)	0.182 (0.075)	0.162 (0.048)	0.165 (0.048)	0.162 (0.045)	0.147 (0.041)*	0.133 (0.041)**	0.117 (0.037)**	0.186 (0.060)**	0.203 (0.073)**	0.237 (0.079)**
Kobayashi [33]		Flexion moment impulse: (Nm·s/kg)	-0.009 (0.011)	-0.007 (0.008)	-0.007 (0.010)	-0.007 (0.015)	-0.009 (0.011)	-0.009 (0.009)	-0.008 (0.007)	-0.011 (0.010)	-0.015 (0.013)**	-0.018 (0.015)**	**-0.007 (0.009)**	-0.005 (0.008)*	-0.004 (0.007)*
		Minimum moment: (Nm/kg)	-0.180 (0.136)	-0.155 (0.141)	-0.145 (0.166)	-0.107 (0.142)**	-0.212 (0.135)	-0.214 (0.134)	-0.226 (0.121)	-0.219 (0.131)**	-0.243 (0.144)**	-0.274 (0.157)**	-0.159 (0.140)*	-0.138 (0.138)**	-0.113 (0.136)**
		Moment at 45% of stance phase: (Nm/kg)	0.245 (0.155)	0.231 (0.141)	0.202 (0.135)	0.163 (0.197)	0.277 (0.150)	0.306 (0.137)*	0.319 (0.104)*	0.240 (0.120)	0.182 (0.145)	0.157 (0.150)*	0.298 (0.149)**	0.318 (0.165)**	0.363 (0.143)**
*Continues from previous page*		Maximum moment: (Nm/kg)	0.830 (0.099)	0.837 (0.124)	0.838 (0.127)	0.850 (0.138)	0.799 (0.121)**	0.750 (0.107)**	0.698 (0.119)**	0.742 (0.102)**	0.712 (0.088)**	0.694 (0.090)**	0.855 (0.119)	0.877 (0.134)	0.905 (0.134)*
				Abduction			Adduction			Medial translation			Lateral translation		
Kobayashi [26]	Orthocare Innovation cohort, 2014	**Coronal (- = varus)**		2°	4°	6°	2°	4°	6°	5mm	10mm	15mm	5mm	10mm	15mm
		Valgus moment impulse: (Nm·s/kg)	-0.003 (0.004)	0.001 (0.001)*	0 (0)	0 (0)	0.011 (0.009)	0.028 (0.019)**	0.053 (0.028)**	0.007 (0.007)*	0.018 (0.014)**	0.034 (0.022)**	0.001 (0.002)*	0 (0)	0 (0)
Kobayashi [33]		Varus moment impulse: (Nm·s/kg)	-0.028 (0.017)	-0.058 (0.026)**	-0.086 (0.024)**	-0.113 (0.042)**	-0.011 (0.011)**	-0.004 (0.005)**	-0.002 (0.004)**	-0.015 (0.011)**	-0.006 (0.005)**	-0.002 (0.002)**	-0.053 (0.027)**	-0.074 (0.027)**	-0.105 (0.026)**
		Moment at 30% of the stance phase: (Nm/kg)	-0.081 (0.064)	-0.149 (0.076)**	-0.193 (0.087)**	-0.246 (0.126)**	-0.023 (0.056)**	0.031 (0.056)**	0.084 (0.061)**	-0.043 (0.075)**	0.009 (0.065)**	0.057 (0.071)**	-0.130 (0.081)**	-0.184 (0.092)**	-0.225 (0.093)**
		Moment at 75% of the stance phase: (Nm/kg)	-0.046 (0.082)	-0.104 (0.089)**	-0.148 (0.088)**	-0.208 (0.092)**	-0.001 (0.088)**	0.054 (0.101)**	0.108 (0.110)**	-0.018 (0.080)**	0.023 (0.088)**	0.068 (0.085)**	-0.101 (0.095)**	-0.129 (0.090)**	-0.187 (0.091)**

*Abbreviations*: *kg = kilogram*, *mm = millimetre*, *Nm/kg = Newton metre*, *N/S = Not significant*, *s* = second, SD = Standard Deviation

* Significant difference (p<0.05) between nominal alignment and malaligned condition

** Significant difference (p<0.01) between nominal alignment and malaligned condition

Six of the included studies had an affiliation with Orthocare Innovations (OI) and presented data from two different cohorts: four studies obtained data from the ‘OI 2012’ cohort [27,28,30,31] and two studies the ‘OI 2014’ cohort [26,33]. An additional two studies [23,24] had an affiliation with ‘Centre de Readaptations Louis Pierquin’ and presented data obtained from the same cohort, which will be called the ‘CRLP’ cohort in this review. The remaining studies [25,29,32] appear to be based on unique cohorts.

### Experimental Procedures

Two of the included studies[23,24] started from an alignment where no alternations had been made on the participant’s own prosthesis. In eight studies [25–31,33] a visual dynamic alignment of the prosthesis of all participants was executed by experienced prosthetists before starting the measurements, and was called the nominally or initial alignment. Given that both nominally and initial alignment are one and the same starting point, this review only used nominal alignment. One study [32] performed a static nominal alignment using the LASAR-posture® -system [34].

Alignment perturbations are reported as change of orientation of the foot in relation to the socket or vice versa. Three studies investigated the influence of transverse alignment changes by rotating the foot relative to the socket [23–25]. The studies of the CRLP cohort compared the nominal alignment to a 6° external rotation and a 6° internal rotation [23,24]. One study compared the nominal alignment to alignment changes of +18° and +36° external rotation [25].

Seven studies [26–31,33] included sagittal and coronal alignment changes on the socket relative to the foot. The studies of the OI 2012 cohort [27,28,30,31] made adjustments on the nominal alignment by 3°/6° flexion/extension/abduction/adduction and 5mm/10mm anterior/posterior/medial/lateral translation. These changes are consistent with the adjustments applied on the OI 2014 cohort: 2°/4°/6° flexion/ extension/abduction/adduction and 5mm/10mm/15mm anterior/posterior/medial/lateral translation. Larger adjustments have been investigated by Pinzur *et al*. with a change of the nominal alignment by 10° flexion/extension/abduction and adduction of the socket relative to foot [29].

Schmalz *et al*. applied all alignment changes in the sagittal plane. They changed the foot relative to the socket by: 2cm anterior and posterior translation and 10° dorsiflexion and plantar flexion of the foot [32].

With the exception of Schmalz *et al*. [32], all the studies used a self-selected walking speed for the reported outcome measurements [23–31,33]. This single study of Schmalz *et al*. performed measurements at two pre-set speeds: a walking speed of 4.0 km/h for five minutes directly followed by 4.8 km/h for five minutes [32].

### Spatiotemporal gait parameters

The extracted spatiotemporal gait parameter data from the included studies are shown in Table 3. Three studies [23–25] included the influence of the transverse plane adjustments by rotating the foot relative to the socket. These studies also compared the PL to the NPL in different alignment conditions.

#### Transverse plane

The studies of the CRLP cohort [23,24] reported a significant longer single support phase and a higher percentage of the stance phase during the gait cycle at the NPL compared to all three PL conditions: nominal alignment, 6° internal rotation and 6° external rotation. Moreover, Fridman *et al*. reported a significantly larger step length for all three conditions of the PL (nominal alignment, +18° and +36° external rotation), compared to the NPL [25].

Fridman *et al*. demonstrated a significant decrease of leg symmetry between PL and NPL at +36° condition, for stance time, swing time and step length, compared to the nominal alignment condition. The PL stance time was significantly shorter than the stance time on the NPL and the PL swing time was significantly longer compared to the NPL. An increase in external rotation resulted in a significant decreased step length for both the PL and NPL. No significant differences have been found in spatiotemporal gait parameters in the +18° external rotation condition [25].

Additionally, both studies of the CRLP cohort included the effect of internal rotation and reported a significant decrease in single limb support phase of the PL, compared to the nominal alignment [23,24].

#### Sagittal plane

Seven studies [26–29,31–33] investigated the influence of sagittal plane adjustments on the spatiotemporal gait parameters. One study [32] investigated the influence on velocity and step length after sagittal plane adjustments and reported no significant change in any alignment condition compared to the nominal alignment. Pinzur *et al*. also did not report a significant difference compared to the nominal alignment when evaluating the stance phase time [29]. Both OI 2012 cohort studies [27,31] investigated cadence and reported a significant decrease after 3°/6° extension and 6° of flexion, compared to the nominal alignment. Furthermore, a significant positive correlation of the maximum sagittal ESRM to cadence was reported, where an increased cadence induced an increased ESRM [28].

#### Coronal plane

Six studies investigated the influence of coronal plane adjustments on the spatiotemporal gait parameters [26–29,31,33]. Pinzur *et al*. [29] and the OI 2014 cohort studies [26,33] did not report a significant change of the spatiotemporal gait parameters after alignment perturbation, compared to the nominal alignment. This is in contrast to three studies using the OI 2012 cohort, where two [28,31] reported a significant reduction in cadence at an alignment of 3° adduction, compared to the nominal alignment. The remaining study [27] from the OI 2012 cohort reported an earlier occurrence of the maximum varus/valgus ESRM during stance phase after the alignment changes of 3°/6° adduction and 10mm medial translation, compared to all other alignment conditions.

### Kinematic gait parameters

All kinematic gait data extracted from the included studies are shown in Table 4. Three studies [23–25] included kinematic gait parameters in their research and made adjustments in the transverse plane by rotating the foot relative to the socket. The studies using the CRLP cohort measured a significant change in the foot progression angle, more externally orientated at external rotation condition and more internally orientated at internal rotation condition [23,24]. In contrast, Fridman *et al*. did not include internal rotation however, they adjusted the nominal foot alignment to an extra 18° and 36° external rotation, which resulted in a smaller external rotation than the expected sum [25]. Consequently, the difference of the external foot rotation between PL and NPL increased significantly in both conditions compared to nominal alignment.

A single study included knee kinematics and reported a significant decrease of the maximal knee flexion angle at the PL during internal rotation condition compared to both the nominal alignment and the control group. The maximal knee flexion of the NPL was significantly higher at the internal rotation condition, compared to the nominal alignment, PL and control group [24].

The hip kinematics after alignment adjustments was investigated in Grumillier *et al*. At the PL the sagittal range of motion (ROM) during 0–8% of the gait cycle appeared significantly more extension-orientated regardless of the alignment condition, compared to the control group. During this initial gait cycle phase (0–8%) the NPL hip was significantly more flexion-orientated, compared to the control group and the PL conditions in the TTA group. Internal rotation of the foot resulted in a significantly more flexion-orientated NPL ROM at 0–8% of the gait cycle compared to the ROM of the NPL during nominal alignment. The same study investigated the hip ROM during 8–30% of the gait cycle and concluded a significantly increased extension-orientated ROM at all three conditions of the NPL, compared to all PL conditions and the control group [23].

Grumillier *et al*. also investigated the occurrence of maximal hip flexion during the gait cycle. The maximal hip flexion at the PL occurred before initial contact of the PL and that of the NPL after initial contact of the NPL. Additionally, a significant increase of the maximal knee flexion at the NPL was observed at internal rotation-condition, compared to the nominal alignment and the control group [23].

### Kinetic gait parameters

The kinetic gait data extracted from the included studies is shown in Table 5. A total of six studies included kinetic data, divided into two cohorts: the OI 2012 and OI 2014 cohort.

Of the six studies, five studies (concerning two cohorts) reported the influence of an alignment adjustment in a given plane on the kinetics in that same plane (e.g. the influence of sagittal alignment adjustments on sagittal kinetics) [26–28,30,33]. Kobayashi *et al*. [27] and Kobayashi *et al*. [28] both focus on a graphical interpretation of the OI 2012 cohort whereas Boone *et al*. investigated the quantitative interpretation of the same cohort [30]. Therefore this review’s kinetic section will focus on three studies: one of the OI 2012 cohort [30] and two of the OI 2014 cohort [26,33].

Only one study investigated [31] the out-of-plane socket ESRM’s, the influence of sagittal alignment on the coronal plane and vice versa.

#### Sagittal plane

Two studies [30,31] of the OI 2012 cohort and one study [26] of the OI 2014 cohort investigated three parameters in the sagittal plane: the maximal and minimal ESRM and ESRM at 45% of stance phase. The other study of the OI 2014 cohort investigated the sagittal ESRM’s impulse [33].

A significant reduction of the minimal sagittal ESRM has been reported in the OI 2012 cohort after an adjustment by: 3° flexion, 6° flexion and 5mm and 10mm posterior translation, compared to nominal alignment. Similarly, a significant reduction of the minimal sagittal moment after alignment perturbations of 6° flexion and 5/10/15mm posterior translations, compared to the nominal alignment was reported by Kobayashi *et al*. [26] (OI 2014 cohort). Additionally, Kobayashi et al. reported an increase of the sagittal minimum ESRM after 5mm/10mm/15mm anterior translation [26].

A significant decrease of the maximal sagittal ESRM (OI 2012 cohort) has been reported after 6° extension and 10 mm anterior translation [30]. Similarly, a 2°/4°/6° extension as well as a 5/10/15mm anterior translation induced the same results for Kobayashi *et al*. [26] (2014 cohort). The latter also reported a larger maximal sagittal ESRM after an alignment perturbation of 15mm posterior translation, whereas two earlier studies on the OI 2012 cohort had reported the same results following a smaller (5mm/10mm) posterior translation.

A significantly more extension-orientated ESRM at 45% of the gait has been reported on the OI 2012 cohort by Boone *et al*., after an alignment perturbation of 3° and 6° extension [30]. Likewise, Kobayashi *et al*. (OI 2014 cohort) reported the same for five alignment perturbations: 4°/6° extension and 5mm/10mm/15mm posterior translation [26].

#### Coronal plane

Three studies reported the coronal ESRM, two [30,31] investigating the OI 2012 cohort and one [26] investigating the OI 2014 cohort. In both cohorts each coronal alignment perturbations induced a significant change of the coronal ESRM at 30% and 75% of the stance phase. One of the studies on the OI 2014 cohort [33] investigated the ESRM impulse, measured in the coronal plane.

At 30% of the gait the ESRM was significantly more varus-orientated after abduction and lateral translation changes for both cohorts. The ESRM was significantly less varus-orientated after 3° adduction and 5mm medial translation in the OI 2012 cohort and after 2° adduction and 5mm medial translation in the OI 2014 cohort. A sign change is reported on both cohorts after alignment perturbations greater than 3° adduction and 5mm translation, respectively from varus- to a valgus-orientated ESRM [26,30].

At 75% of the gait the ESRM at the nominal alignment was valgus-orientated for the OI 2012 cohort and all perturbations by adduction and medial translations induced a significantly larger valgus moment. Additionally, a significant change from valgus to varus was reported at 75% of the gait after all abduction and lateral translation perturbations [30]. With respect to the OI 2014 cohort [26], a varus-orientated ESRM was reported at nominal alignment, where 2° adduction and 5mm medial translation produced a significantly smaller varus ESRM. Larger perturbations in adduction and medial translation resulted in a significant change from varus to valgus. Moreover, significantly larger varus moments were reported after all abductions and lateral translations.

Only Kobayashi *et al*. (2014 cohort) investigated the ESRM impulses, separated into valgus impulse (0–30% of stance phase) and the varus impulse (30–100% of stance phase) [33]. Only seven coronal perturbations, 2°abduction, 4°/6° adduction, 5mm lateral translation and 5mm/10mm/15mm medial translation, influenced the valgus ESRM impulse during gait. All 12 coronal alignment perturbations produced a significant change in the varus ESRM impulse[33].

Furthermore, Kobayashi *et al*. [31] investigated the influence of sagittal alignment changes on the coronal ESRM was studied. A significant reduction of the ESRM at 30%, after sagittal perturbations of 3° and 6° extension, was found.

## Discussion

In this review, the effects of alignment changes in coronal/sagittal and transverse direction have been assessed. Changes in the transverse plane demonstrated a clear effect on foot progression angle and internal hip rotation angle. Coronal and sagittal plane adjustments showed an important influence on external socket reaction moments and impulses. A remarkable result from this review is the observation that a predetermined alignment change did not result in a predictable kinematic or spatiotemporal change.

This systematic review’s results report no considerable changes in kinematic and spatio-temporal gait data after alignment changes. These observations suggest that patients with a TTA appear to minimize the effect of alignment changes by kinetic adaptions. For example, a rotation change of the prosthetic foot in the transverse plane does not result in a predictable change of the foot progression angle during gait [23–25]. It appears that TTA patients compensate for the latter alignment changes by additional internal hip rotation. While at first the alignment is satisfactory for both the prosthetist and amputee, since optical gait observation suggests a correct alignment, it may induce stump problems later on during more extended walking periods [9,22]. In order to produce consistent spatio-temporal and kinematic gait parameters adjustments are required in the TTA’s kinetic parameters. This is supported by this review, since alignment alternations result in a predictable change in kinetic data, reported as ESRM’s.

Interestingly, kinetic parameters are also subjected to compensatory mechanisms. A considerable alignment change of 4°/6° abduction and 10/15mm lateral translation in the OI 2014 cohort did not produce a significant change in the ESRM impulse, compared to nominal alignment [33]. However, consistent significant changes are reported for ESRM parameters after smaller alignment changes in the same study. Additional muscular forces may have been induced to change kinetic force patterns on the lower limbs to obtain a more comfortable magnitude.

It is not known what an ideal moment and impulse should be in each phase of the gait. The OI 2012 and OI 2014 cohort studies describe the nominal alignment as a standardized position and determine a perturbations’ influence significance by a subjective starting point. It could be expected that the timings of ESRM measurement be in the same order of timing as the moments working in the same plane on the knee joint. However, the magnitude of the moments will differ at the knee joint compared to the base of the socket. Whereas the magnitude of the ground reaction force is the same, the distance for the calculated moment around the base of the socket is likely different from the distance to the knee joint centre.

The ESRM is influenced differently by angulations and translations. In the included studies, angulations changes have a slightly bigger influence on ESRM compared to translations. This may be expected where an angulation in the coronal plane is producing both a translation and a foot eversion (or inversion) and a sagittal angulation will induce a translation and a foot plantar flexion (or dorsiflexion). Therefore, it may be interesting to investigate the changes separately or present the flexion/extension and abduction/adduction data as the change of foot position relative to the socket. Study data should be consistently presented to allow comparison of studies in meta-analysis and consensus evaluations, which would help to improve the analysis of an optimal alignment approach.

Although most studies examined the effect of alignment changes in a given plane on kinematics and kinetics in the same plane, one study focused on out-of-plane effects. Kobayashi *et al*. reported an effect of sagittal alignment angulation on coronal kinetic data and vice-versa [31] The findings may have important implications for the clinical alignment process. These results imply that it is preferable to start the alignment process with transverse plane adjustments, influencing sagittal and coronal parameters, followed by sagittal adjustments and ending with coronal adjustments [31]. However, since only one study to date has reported out-of-plane adjustments, further research is required to evaluate these effects in detail.

At present, there is no golden standard to achieve an optimal prosthetic alignment, since alignment is performed on an individual basis, where the end-point resulting in a perfect fit is unknown. An indication of a correct prosthetic alignment may be provided by an increase in symmetry between the PL and NPL. However, increasing gait symmetry for TTA does not necessarily result in an improvement of gait [4]. Moreover, asymmetry may even be a prerequisite to achieve gait stability during ambulation [1]. Reported studies question if current prosthetic alignment approaches are the correct method to optimize prosthetic alignment, since the current approaches are based on achieving symmetry in spatiotemporal and kinematic gait data [35,36].

A limitation of prosthetic alignment research is that gait deviation can be related to prosthetic socket-fit as well as to prosthetic alignment. Ideally, the socket-stump interface is as stiff a coupling as the normal abled-bodied leg. This is not the case, as such, movement inside a socket will result in unwanted error at alignment measurements through the resulting stump volume change, improper use of stump socks or an insufficient prosthetic fit. It is especially interesting regarding this review, and for future alignment studies, to find a way to quantify both alignment and prosthetic fit. Moreover, socket fit and alignment are potentially interlinked, where alignment could provide an approach to solve socket-fit mismatches.

Several interesting results are seen in the critical appraisal results (Table 2). Most of the appraised studies did not adequately describe the patient population, of which only one provided a clear description of the study sample. It is therefore advised to expand the methodology sections to properly describe the study’s in- and exclusion criteria and to include a detailed baseline table in original research articles. Which should include items such as: age, weight, height, gender, level of amputation, residual limb length, reason for amputation, activity level of the amputee, time since onset and stump condition. Additionally, an improvement on controlled interventions is desirable with a minimum of variables influencing the outcome measurements. For example, a considerable amount of research is executed on different prosthetic components, e.g. prosthetic feet, shoes, socket fittings. It is highly recommended that prosthetic research aims towards an overall improvement of the methodology in addition to the use of quantifiable outcome measurements.

One limitation of this systematic review is potentially having missed relevant literature on the chosen subject and might be subjected to selection bias through the exclusion of articles other than the English language. Although a reference check did not result in new studies, the possibility of having missed relevant literature cannot be excluded. Another limitation is the lack of published standardized critical appraisal available for the chosen subject. A validated scoring system would have helped to better identify methodologically sound, or potentially less strong, data. However, an adjusted critical appraisal in addition with the validated COSMIN checklist led to a clear view on the methodological quality of the included articles.

The results described in this review highlight that alignment changes have a consistent influence on the kinetic parameters: ESRM and ESRM impulse. However, individuals may respond differently towards alignment adjustments [28]. The results suggest that an acceptable range, instead of an exact value, may be established as an ideal alignment criteria, where the range can be varied depending on the function and perception of the individual. [27]

## Conclusion

Prosthetic alignment changes have no consistent influence on spatio-temporal and kinematic gait data. This is in contrast to kinetic parameters that show significant clinical relevant changes after alignment adjustments. It is highly recommended that prosthetic research should aim towards an overall improvement of the study design. The findings from this review suggest that it is imperative to include kinetics in the process of dynamic prosthetic alignment. While current literature is not conclusive about an optimal alignment, future alignment research should focus on alignment optimization based on kinetic outcomes.

## Supporting Information

S1 Checklist PRISMA 2009 Checklist. (DOC)

S1 Appraisal Critical Appraisal. (DOCX)

## Abbreviations

CRLP: Centre de Readaptations Louis Pierquin
ESRM: External Socket Reaction Moment
NPL: Non-Prosthetic Limb
OI: Orthocare Innovations
PL: Prosthetic Limb
ROM: Range of Motion
TTA: Transtibial Amputee
