# Correction: The Effect of Alignment Changes on Unilateral Transtibial Amputee's Gait: A Systematic Review

**DOI:** 10.1371/journal.pone.0188545

**Published:** 2017-11-17

**Authors:** Niels Jonkergouw, Maarten R. Prins, Arjan W. P. Buis, Peter van der Wurff

The fourth author’s name appears incorrectly in the citation. The fourth author in the citation should be van der Wurff P. The correct citation is: Jonkergouw N, Prins MR, Buis AWP, van der Wurff P (2016) The Effect of Alignment Changes on Unilateral Transtibial Amputee’s Gait: A Systematic Review. PLoS ONE11(12): e0167466. https://doi.org/10.1371/journal.pone.0167466
