# Supplementary material for: The Effect of Alignment Changes on Unilateral Transtibial Amputee’s Gait: A Systematic Review
Source: PLoS One. 2016 Dec 6;11(12):e0167466. doi: 10.1371/journal.pone.0167466 (PMC5140067; doi:10.1371/journal.pone.0167466)
Supplement: S1 Appraisal — (DOCX) [file pone.0167466.s002.docx]

# Critical Appraisal

**Selection of Patients**

**A1**

Adequacy of Description of Inclusion and Exclusion Criteria: This criterion tested whether the patient sample was sufficiently defined with the use selection criteria: such as age, gender, level of amputation, reason for amputation, activity level of the amputee, time since onset, stump condition and comorbidity. This item was scored with a ‘1’ when not more than one of the previous mentioned criteria are missing.

**A2**

Functional Homogeneity: A clear description of the study sample was provided. Age, height, weight, gender and cause of amputation, residual limb length and activity level should have been described to obtain a “1 score”.

**Intervention and Assessment**

**B3**

Experimental Intervention: The experimental intervention had to be described in such detail as to make performing a duplicate study, as described, possible.

**B4**

The sequence of tests should be at random: the alterations in prosthetic alignment had to be imposed in a quasi-random order to control for order effects.

***B5A***

Familiarisation time initial alignment: There should be a clear description of the amount of time the amputee had worn the foot and tube and their relative alignment that was used during the experiment (e.g. less than an hour, less than a day, less than two weeks or over two weeks).

**B5B**

Familiarisation time alternative alignments: A clear description of the amount of time available for the amputee to adapt to each alignment perturbation.

**B6**

Outcome Measures: The measurement tools should be adequately described in relation to the purpose of the study and outcome measurements, and they should have been collected with the use of a standardized protocol.

**Statistical validity:**

**C7**

Sample Size: The sample size should be at least 10 individuals.

**C8**

Data Presentation: This criterion required that the primary outcome measurements were presented with mean values and a standard deviation.

**C9**

Were all the statistical tests described clearly?
